# Measuring Negative Attitudes towards Overweight and Obesity in the German Population – Psychometric Properties and Reference Values for the German Short Version of the Fat Phobia Scale (FPS)

**DOI:** 10.1371/journal.pone.0114641

**Published:** 2014-12-04

**Authors:** Janine Stein, Melanie Luppa, Ulrike Ruzanska, Claudia Sikorski, Hans-Helmut König, Steffi G. Riedel-Heller

**Affiliations:** 1 Institute of Social Medicine, Occupational Health and Public Health, Faculty of Medicine, University of Leipzig, Leipzig, Germany; 2 Institute for Psychology, Faculty of Bioscience, Pharmacy und Psychology, University of Leipzig, Leipzig, Germany; 3 Leipzig University Medical Centre, Integrated Research and Treatment Centre (IFB) Adiposity Diseases, University of Leipzig, Leipzig, Germany; 4 Department of Health Economics and Health Services Research, Hamburg Centre for Health Economics, University Medical Centre Hamburg-Eppendorf, University of Hamburg, Hamburg, Germany; Old Dominion University, United States of America

## Abstract

**Objective:**

Obesity is one of the leading public health problems worldwide. Obese individuals are often stigmatized and the psychosocial consequences of overweight and obesity are the subject of current research. To detect stigmatizing attitudes towards obese people, the Fat Phobia Scale (FPS) was developed in the USA in the early nineties. In addition, the 14-item short form of the FPS was constructed. The FPS belongs to the most commonly used instruments for measuring negative attitudes towards obese people because of its good psychometric properties. For the recently developed German short form of the FPS, however, the comprehensive investigation of the psychometric properties and the determination of reference values are still pending. Thus, the main objectives of this study were the evaluation of the psychometric quality of the scale as well as the calculation of reference values.

**Methods:**

The study was based on a representative survey in the German general population. A sample of 1,657 subjects (18–94 years) was assessed via structured telephone interviews including the 14-item German version of the FPS. Descriptive statistics and inference-statistical analyses were conducted. Reference values in terms of percentage ranks were calculated.

**Results:**

Substantial evidence for the reliability and validity of the German short version of the FPS was found. This study, for the first time in Germany, provides age-specific reference values for the German short form of the FPS allowing the interpretation of individual test scores.

**Conclusion:**

Facing the far-reaching consequences of experienced stigmatization of obese individuals, these study results provide an important basis for further studies aiming at the investigation of negative attitudes towards overweight and obesity.

## Introduction

Overweight and obesity represent one of the most challenging health and societal problems of the 21^st^ century. In 2008, the World Health Organisation (WHO) estimated that 1.5 billion adults (35%), aged 20 years and older, were overweight and of these over 200 million men and nearly 300 million women were obese. Worldwide, obesity prevalence rates are rising [1], [2]. Many studies showed that obese individuals may not only suffer from serious physical health consequences (e. g. diabetes, cardiovascular disease, hypertension or dyslipidemia) but also may be negatively affected by social problems and restrictions that may occur because of negative attitudes towards and discrimination of obese people. Consequently, stigmatization in the workplace, in healthcare, in the educational sector and in the media may occur [3]–[7].

Considering the prevalence of obesity and the stigmatization faced by obese people, the use of appropriate instruments for measuring negative attitudes towards obese individuals is indispensable. Besides the assessment of the extent of stigma against obese people these instruments are also essential for the evaluation of interventions to reduce stigma. In this context, only instruments that fulfill the psychometric quality criteria (objectivity, reliability, validity) should be used and the evaluation of the used instruments is of particular importance. In addition, the establishment of up-to-date reference values based on large and representative samples is highly desirable.

Morrison, Roddy & Ryan (2009) [8] provided an overview of the main instruments for measuring negative attitudes towards obese people that have been developed since 1995 including the Fat Phobia Scale (FPS). The FPS was developed and introduced by Robinson, Bacon & O’Reilly (1993) [9] in the USA. Bacon, Scheltema & Robinson (2001) [10] developed a shortened 14-item version of the FPS by extracting the first factor of the 50-item original FPS scale. The psychometric properties of the scale were investigated and proved in several studies [4], [9], [11], [12]. According to Yuker, Allison & Faith (1995), who compared 23 different methods for the measurement of attitudes towards overweight, the FPS represents one of the best four instruments for the assessment of attitudes towards obese people due to its good psychometric properties [13].

In the present study, the short form of the FPS was used which had been translated into the German language by Sikorski et al. (2012) [14] following the TRAPD guidelines (Translation, Review, Adjudication, Pre-Testing and Documentation) [15], [16]. First analyses showed a good reliability coefficient of α = 0.79 for the German short version [14]. However, comprehensive analyses on the psychometric quality of the German version of the FPS have not yet been performed. Moreover, Bacon et al. (2001) [10] particularly emphasized that reference values on the FPS for the general population need to be established. Against this background, the aims of this study were to (1) investigate the psychometric quality (reliability and validity) of the German short version of the FPS and (2) to calculate reference values for the scale based on a large sample that is representative for the German population.

## Materials and Methods

### Study Design and Subjects

Analyses were based on a sample of individuals who participated in the large, population-based project “The Stigma of Overweight and Obesity in the General Population and Among Health Care Professionals”. The project primarily investigated attitudes of the German population (General Population Survey GPS, 18+ years) towards obesity and assessed stereotypes and social stigma associated with obesity. The survey was conducted as a computer-assisted telephone interview (CATI) by USUMA, a leading market, opinion and social research institute in Germany. Data was collected from February to April 2011. Sampling was based on random digital dialing, drawing from the Association of German Market and Social Research Agency’s (ADM) sample base that includes registered and non-registered telephone numbers. Representativeness of the study sample to the German population has been ensured by including all regions in Germany in the sampling process. The Kish-Selection-Grid was applied when randomly selecting the person in the household (at least 18 years of age) to be interviewed to ensure equal probability of participation for each member of the randomly selected household [17]. A total of 5,897 individuals were randomly selected and contacted from which 1,998 (32.4%) refused to participate in the study. 16.5% of the selected individuals were not available. The response rate was 50.9% and the overall sample consisted of n = 3,003 individuals. Data were gathered by using a standardized structured interview. All interviewers were trained for conducting the interview by members of the study team. The design of the study has been described in detail by Sikorski et al. [14]. All analyses of this study are based on a sample of 1,657 individuals. In order to obtain this number, 1,002 participants of initially 3,003 respondents were excluded from the study sample because they have been asked to rate the children vignette and this study focused on negative attitudes towards overweight and obese adults and seniors. Furthermore, 344 participants with incomplete and missing values on the FPS (overweight vignette) were excluded from analyses.

### Ethics Statement

The study was approved by the Ethics committee of Leipzig University (Ethik-Kommission an der Medizinischen Fakultät der Universität Leipzig). All participants gave verbally informed consent. The consent and refusal of each participant was documented by USUMA, the conducting market research institute within the computer-assisted interview. The ethics committee specifically approved this procedure.

### Procedure and Instruments

The survey was conducted by USUMA using standardized questionnaires. The socio-demographic variables age, education/occupation, gender, height/weight, marital status, level of income and migrational background were assessed. The questionnaire included the German version of the Fat Phobia Scale (FPS) [10], [14]. The German version of the FPS can be obtained from the authors. This short version of the scale contains 14 pairs of adjectives on a semantic differential assessing negative attitudes and fat phobia towards overweight or obese individuals. For each item pair, the subject is asked to choose the position on a scale from 1 to 5 closest to the adjective that best describes obese or fat people. For the original short form of the FPS, Cronbach’s α was found to range between α = 0.79 and α = 0.91 reflecting a good to excellent reliability. Validity of the original scale was proved, for example, in the study of Robinson et al. (1993) [9]. In this study, vignettes describing an obese individual varying in gender (female/male), and age (42-year-old adult and 68-year-old senior citizen) were used. In all cases, the person in the vignette was described and introduced as strongly overweight. The structured interview also contained the Social Distance Scale (SDS), which was developed by Bogardus (1933) [18] and modified by Link et al. (1987) [19]. The scale consists of 7 sentence descriptions representing different types of social relationships or situations (sub-letting, neighborhood, common place of work, personal job brokering, marriage into one’s family, member of the same social circle, child care). The subjects were asked to rate if they would accept the person described in each relationship or situation on a scale from 1 (“in no case at all”) to 5 (“in any case”) according to the amount of social distance each acquired. In order to assess the affective components of negative attitudes towards overweight or obese individuals, a list of 7 Likertscaled items ranging from 1 (definitely the case) to 5 (definitely not the case) was used to assess emotional reactions to the individual described in the vignette. The items of this scale (SER scale) represent three ways to respond to mentally ill people: fear, pity and anger [20]. Furthermore, a modified 6-item version of the Perceived Devaluation and Discrimination Scale (PDD) [21] was used to assess the cognitive components of negative attitudes towards overweight or obese individuals. The participants were asked to rate statements targeting at common prejudices of the general population towards overweight or obese individuals on a scale ranging from 1 (fully disagree) to 5 (fully agree). In the last part of the questionnaire, the participants were asked to rate the FPS after the presentation of a normal weight vignette describing a normal weight individual varying in gender (female/male), and age (42-year-old adult and 68-year-old senior citizen).

### Statistical analysis

All analyses were performed using SPSS version 20 for Windows. All positive items of the FPS (1, 2, 8, 9, 11 and 13) were recoded. The total score of the FPS was calculated by summing up all 14 items with higher scores indicating higher negative attitudes. The weighted FPS score was build by dividing the total score by the total number of items (14). Analyses on item and scale level were conducted taking into account mean, standard deviation, and distribution of FPS scores according to an established cut-off [14], [22]. FPS scores below 2.49 were defined as mainly neutral or positive attributes and scores above 2.50 indicated mainly negative attributes [14], [23], [24].

Wilcoxon sign-ranked tests (non-parametric statistical hypothesis test) and Student t-tests (two-tailed) were used to investigate significant mean differences in FPS scores on a group level. One-way analyses of variance (ANOVA) were conducted to investigate factors that were significantly associated with the level of FPS score. ANOVA is an extension of the Student t-test used to analyze variation between several group means by comparing variance among groups relative to variance within groups [25]. As done in previous research, age was introduced by dividing the sample into five age groups [14]. Other variables were educational status in years, occupational status, net household income per month, residence (former Eastern part of Germany, Western part of Germany), and overweight in the past (yes/no). The calculated BMI was categorized according to guidelines (underweight <18.5 kg/m^2^, normal weight 18.5–24.9 kg/m^2^, overweight 25.0–29.9 kg/m^2^ and obese >30 kg/m^2^). Mean FPS score was introduced as dependent variable and socio-demographic factors as independent variables.

In order to determine reliability of the FPS, the reliability coefficient in terms of Cronbach’s α (coefficient of internal consistency) was calculated. By definition, acceptable values range from α = 0.70 to 0.95 [26]. Construct validity of the FPS in terms of convergent and discriminant validity (positive or negative correlations between items where one would expect such correlations or a lack of such correlation, respectively) was assessed by investigating the associations between the total score of FPS and other related or unrelated instruments (SDS, SER, PDD) via correlations (Spearman’s ρ). For the purpose of assessing the dimensionality of the FPS scale, a factor analysis (principal components analysis, varimax rotation) was performed. If not otherwise stated, level of α-error was set to 0.05 for all computations. Finally, reference values in terms of percentage ranks were calculated for the total sample as well as for different age groups allowing the classification and interpretation of FPS scores for the German population.

## Results

### Socio-demographics

Table 1 provides an overview of the characteristics of the study sample (n = 1,657). Mean age of participants was 51.3 years ranging from 18 to 94 years. Approximately half of the sample (47.7%) was female. Nearly half of the participants were of normal weight (48.5%) or overweight (49.3%). 958 participants (57.8%) reported that they had experiences with overweight.

**Table 1:** Socio-demographic characteristics of the study sample (n = 1,657).

		n	%
Age (in years)			
M (SD)	51.3 (17.8)		
Range	18–94		
Age group			
18–20		90	5.4
21–40		375	22.6
41–60		624	37.7
61–80		517	31.2
80+		51	3.1
Gender			
Female		791	47.7
Male		866	52.3
Level of school education			
Student		21	1.3
8/9 years of schooling		396	23.9
10 years of schooling		560	33.8
12/13 years of schooling		671	40.5
No school graduation		5	0.3
No information		4	0.2
Occupational status			
Employed		792	49.0
Student or trainee/apprentice		146	7.5
Draftee/community service orvoluntary social/ecological year		3	0.2
Unemployed		56	3.4
Housewife/houseman		87	5.3
Retirement/early or partial retirement		558	33.7
Disability/invalidity pension		9	0.5
Maternity/parental leave		6	0.4
Net household income per month^1^			
<1000		128	7.7
1000<2000		521	31.5
2000<3000		440	26.5
3000+		365	22.0
No information		203	12.3
Residence			
Western part of Germany		1,369	82.6
Former Eastern part of Germany		288	17.4
Body mass index (BMI)			
<18.5		28	1.7
18.5–24.9		804	48.5
25.0–29.9		580	35.0
>30		238	14.4
No information		7	0.4
Overweight in personal history			
Yes		958	57.8
No		699	42.2

Notes. M = Mean, SD = Standard deviation, ^1^in Euro.

### Analyses on scale and item level

As displayed in table 2, the mean FPS score (i.e. the stigma index) for the overweight vignette in the total sample was 3.62 (SD = 0.49) and 2.37 (SD = 0.47) for the normal weight vignette. Wilcoxon test for paired samples showed that the differences between the FPS scores in both vignette conditions were highly significant (Z = −34.66, p<0.001) indicating a higher level of negative attitudes towards overweight and obesity. Stigma difference index was calculated by subtracting the mean FPS score of the normal weight vignette from the mean FPS score of the overweight vignette [27]. The stigma difference index was 1.25 indicating that on average the negative attitudes towards obese individuals were 1.25 points higher than towards normal weight individuals. Overall, distribution of FPS scores differed significantly from normal distribution in both vignette conditions (table 2). Furthermore, negative attitudes towards obese individuals according to the cut-off of 2.5 with FPS ≤2.49 indicating neutral or positive and FPS ≥2.5 indicating negative attitudes towards obese individuals were analyzed. In the condition of the overweight vignette, 99.1% of the participants showed negative attitudes and only 0.9% showed neutral or positive attitudes towards obese individuals. In contrast, 47.7% of the participants in the condition of the normal weight vignette showed negative attitudes and 51.9% showed neutral or positive attitudes towards obese individuals. All results are summarized in table 2.

**Table 2:** Mean scores of Fat Phobia Scale (FPS) and distribution of FPS scores according to a cut-off indicating neutral/positive or negative attitudes towards overweight and obesity [14], [22].

Vignette	n	M	SD	Range	Skewness	Kurtosis	Z
Overweight	1,657	3.62	0.49	1.86–5.00	0.17	0.15	2.53***
Normal weight	1,651	2.37	0.47	1.00–5.00	−0.33	0.12	3.42***
Cut-off	Neutral or positive (FPS≤2.49)				Negative (FPS≥2.5)		
Vignette	n		%		n	%	
Overweight	15		0.9		1642	99.1	
Normal weight	860^1^		51.9		791^1^	47.7	

Notes. n = Sample size, M = Mean, SD = Standard deviation, Test statistic Z = Kolmogorov-Smirnov-Z, significance level (two-tailed) *p<0.05, **p<0.01, ***p<0.001, ^1^Exclusion of n = 6 participants because of missing values.

Table 3 depicts the item-wise FPS mean scores for both conditions (overweight and normal weight vignettes). For all items, highly significant differences via t-tests were observed meaning that all FPS items in the overweight vignette condition were rated higher than items in the normal weight vignette condition. Additionally, the stigma difference indices were calculated to demonstrate the magnitude of these differences on item level of the FPS scale. The highest differences were observed for the items 5 (fast…slow), 6 (having endurance…having no endurance), 7 (active…inactive) and 11 (shapeless…shapely).

**Table 3:** Analyses on item level of the FPS in different conditions and results of the t-tests (n = 1,657).

		Overweightvignette		Normalweightvignette^1^			
	Pair of adjectives (items)	M	SD	M	SD	Stigma-Difference -Index	t
1	Lazy…industrious	3.22	0.84	2.36	0.85	0.86	28,479***
2	No will power…has willpower	3.58	0.95	2.25	0.89	1.33	38,513***
3	Attractive…unattractive	3.59	0.97	2.27	0.88	1.31	37,745***
4	Good self-control…poorself-control	3.47	0.94	2.32	0.88	1.15	33,390***
5	Fast…slow	3.79	0.99	2.24	0.92	1.55	43,511***
6	Havingendurance…havingno endurance	3.84	1.00	2.12	0.97	1.72	46,810***
7	Active…inactive	3.76	0.94	2.02	0.92	1.74	49,416***
8	Weak…strong	3.31	0.98	2.39	0.91	0.92	25,760***
9	Self-indulgent…self-sacrificing	3.49	0.93	2.58	0.80	0.91	28,589***
10	Dislikes food…likesfood	4.11	0.90	3.32	0.86	0.80	26,874***
11	Shapeless…shapely	3.67	1.13	2.00	0.90	1.67	43,498***
12	Undereats…overeats	4.13	0.87	2.84	0.55	1.29	50,060***
13	Insecure…secure	3.36	0.97	2.25	0.93	1.11	31,680***
14	Low-self-esteem…high self-esteem	3.39	0.95	2.24	0.85	1.15	33,575***

Notes. n = Sample size, M = Mean, SD = Standard deviation, t = test statistic, Significance level (two-tailed) *p<0.05, **p<0.01, ***p<0.001, all significant results are in bold, Items 1, 2, 8, 9, 11 und 13 were recoded, ^1^Exclusion of n = 6 because of missing values.

### Analyses of variance

In table 4, the results of the analyses of variance (ANOVA) are displayed. Analyses revealed that there were significant age effects: younger participants reported higher FPS scores indicating more negative attitudes towards obese individuals than older participants (F = 3.264, df = 4, p = 0.011). Furthermore there were significant differences found for the variable BMI. With regard to the BMI, the results showed that a lower BMI was significantly associated with a higher FPS score and more negative attitudes towards overweight and obesity (F = 16.159, df = 5, p<0.001). For the other variables we did not find any significant differences in FPS scores on a group level. Additional ANOVA (not shown in table 4) revealed that these effects were also found for the stigma difference index (age: F = 9.982, df = 4, p = 0.011; BMI: F = 7.982, df = 5, p<0.001).

**Table 4:** Analyses of mean differences between FPS score and socio-demographic factors – results of the analyses of variance (ANOVA) (n = 1,657).

Variables	Sum ofsquares	df	Mean of squares	F	p
Age	3.204	4	0.801	3.264*	**0.011**
Gender	0.009	1	0.009	0.035	0.852
Education	2.155	4	0.539	2.195	0.067
Occupational status^§^	4.003	10	0.400	1.628	0.093
Net household income	1.733	5	0.347	1.379	0.229
Residence	0.264	1	0.264	1.070	0.301
Body mass index (BMI)^#^	19.025	5	3.805	16.159***	**<0.001**
Overweight in personal history	0.139	1	0.139	0.636	0.426

Notes. df = degrees of freedom, F = test statistic, Significance level (two-tailed) *p<0.05, **p<0.01, ***p<0.001, ^§^Occupational status comprised the categories employed, student, trainee/apprentice, draftee/community or civilian service, unemployed, housewife/houseman, retirement, early or partial retirement, disability/invalidity pension, maternity/parental leave, voluntary social/ecological year, ^#^BMI comprised the categories <18.49, 18.5–24.9, 25–29.9, 30–34.9, 35–39.9, >40.

### Reliability and validity

Cronbach’s α was determined as a measure for internal consistency and reliability of the FPS scale. For the total sample of 1,657 participants Cronbach’s α was 0.791 reflecting a moderate to good reliability of the FPS scale.

In order to determine construct validity of the FPS, the correlations between the FPS score and other construct related scales, namely the Social Distance Scale (SDS), the scale for emotional reactions (SER) and the Perceived Devaluation and Discrimination Scale (PDD), were analyzed. Weak significant positive correlations were observed for the SER and the PDD scales (r = 0.133, p<0.001 and r = 0.150, p<0.001) but not for the SDS (r = −0.045, p<0.097). This means that a higher FPS score (and a higher level of negative attitudes towards obese individuals) was significantly associated with more negative affective/emotional reactions towards overweight or obese individuals as assessed by the SER scale and a stronger agreement with common prejudices of the general population towards obese individuals as assessed by the PDD scale.

In the next step, a factor analysis (principal components analysis, varimax rotation) was performed to determine the dimensionality of the FPS scale. KMO value was 0.872 and test for sphericity according to Bartlett revealed a highly significant result (χ^2^ = 3656.336, df = 91, p<0.001) fulfilling the requirements for the calculation of a factor analysis. Three eigenvalues above the criterion of 1 were observed: (1) 3.819 explaining 27.28% of the variation, (2) 1.248 explaining 8.916% of the variation and (3) 1.071 explaining 7.652% of the variation. As Factor 1 explained 27.28% of the variation, a one factor solution was strongly supported.

### Reference values

Table 5 presents the reference values in terms of percentage ranks for the FPS score (1–70) and for the weighted FPS score (1–5). In order to account for significant age effects, the reference values were calculated not only for the total sample (n = 1,657) but also for different age groups. To give an example, a person aged 77 years reached a FPS score of 58 (weighted FPS score 4.1). According to table 5 (see second last column on the right side, 61–80 yrs), this person reached a percentage rank of 84.1. That means that ≤84.1% persons of the sample reached a FPS score of that magnitude or a lower score. In other words, 84.1% of the general German population in that age group had the same level or a lower level of negative attitudes towards obese individuals.

**Table 5:** Reference values (percentages) for the FPS score and the weighted FPS score for the total sample (n = 1,657) and for different age groups.

	Percentage rank					
FPS score	Total(n = 1,657)	18–20 yrs(n_1_ = 90)	21–40 yrs(n_2_ = 375)	41–60 yrs(n_3_ = 624)	61–80 yrs(n_4_ = 517)	80+yrs(n_5_ = 51)
14						
15						
16						
17						
18						
19						
20						
21						
22						
23						
24						
25						
26	0.1		0.3	0.2		
27	0.2		0.5	0.2		
28	0.2		0.6	0.3	0.2	
29	0.4		0.7	0.3	0.4	
30	0.5		0.8	0.4	0.6	
31	0.5		0.9	0.5	0.7	
32	0.6		1.0	0.6	0.8	
33	0.6		1.1	0.7	0.8	
34	0.9		1.1	0.8	1.2	
35	1.1		1.3	1.1	1.7	
36	1.6		1.6	1.4	2.1	
37	1.8		2.0	1.8	2.7	
38	2.4		2.4	2.1	3.3	2.0
39	3.3		2.9	2.9	4.6	3.9
40	4.7	1.1	4.3	4.5	5.8	5.9
41	6.6	1.7	5.3	7.2	7.2	11.8
42	10.5	2.2	8.8	12.2	10.8	13.7
43	14.5	5.6	12.3	17.0	14.9	17.7
44	19.1	7.8	16.8	21.3	19.7	21.6
45	22.7	10.0	20.8	25.3	23.0	23.5
46	26.7	11.1	24.5	30.1	26.9	27.5
47	33.4	16.7	31.7	38.1	31.7	33.3
48	40.3	27.8	40.8	43.9	37.7	39.2
49	46.4	33.3	47.5	50.2	43.5	45.1
50	51.8	37.8	54.7	55.9	47.8	47.1
51	57.0	43.3	59.7	60.4	53.2	56.9
52	62.6	50.0	64.8	64.3	61.5	60.8
53	67.2	52.2	68.3	69.4	66.2	68.6
54	71.8	57.8	74.4	73.1	70.6	74.5
55	76.0	64.4	79.2	76.8	74.9	76.5
56	79.9	72.2	83.2	80.1	78.3	82.4
57	83.2	76.7	87.5	83.0	81.0	88.2
58	86.9	83.3	90.7	87.0	84.1	92.2
59	89.0	86.7	91.2	90.1	86.1	92.9
60	90.8	87.8	92.5	92.5	88.2	93.5
61	92.3	88.9	94.1	93.6	90.1	94.2
62	94.1	93.3	95.7	94.9	92.3	94.8
63	95.0	96.7	96.3	95.5	93.4	95.5
64	96.4	97.8	97.1	96.8	95.6	96.1
65	97.3	98.9	97.6	97.4	96.5	98.0
66	98.7	100.0	98.4	98.4	99.0	98.7
67	99.0		98.6	98.9	99.4	99.4
68	99.5		98.7	99.5	99.8	100.0
69	99.5		99.9	99.8	100.0	
70	100.0		100.0	100.0		
M	50.73	52.98	50.50	50.31	51.02	50.61
SD	6.955	5.925	6.644	6.940	7.299	6.844
Range	26–70	40–66	26–70	26–70	28–69	38–68
Skewness	0.174	0.140	0.165	0.289	0.072	0.362
Kurtosis	0.152	−0.613	1.062	0.049	−0.089	0.002
WeightedFPS score	Percentage rank					
	Total(n = 1,657)	18–20 yrs(n_1_ = 90)	21–40 yrs(n_2_ = 375)	41–60 yrs(n_3_ = 624)	61–80 yrs(n_4_ = 517)	80+yrs(n_5_ = 51)
1	0.2	0.06	0.5	0.2	3.6	5.9
2	6.6	1.1	5.3	7.2	7.2	11.8
3	76.0	64.4	79.2	76.8	74.9	76.5
4	99.5	100.0	98.7	99.5	100.0	100.0
5	100.0		100.0	100.0		
M	3.62	3.78	3.61	3.59	3.64	3.61
SD	0.49	0.42	0.47	0.50	0.52	0.49
Range	2–5	3–5	2–5	2–5	2–5	3–5
Skewness	0.17	0.14	0.17	0.29	0.72	0.36
Kurtosis	0.15	−0.61	1.06	0.05	−0.09	0.00

Notes. yrs = years, n = Sample size, M = mean, SD = Standard deviation.

## Discussion

The aims of the present study were to evaluate the psychometric quality and the determination of reference values based on a representative population-based sample for the German short version of the Fat Phobia Scale (FPS) for the assessment of negative attitudes towards obese people. To our knowledge, this is the first study in the German-speaking countries to investigate the psychometric properties of the FPS and to provide age-specific reference values for the scale.

### Analyses on scale and item level

Two different stigma indices were calculated that were based on the responses on the two vignettes (overweight and normal weight). The stigma index is defined as the mean FPS score of the evaluation of the obesity vignette. In this study, we found a stigma index (mean FPS score) of 3.62 in the condition of the obesity vignette representing an average level of fat phobia. This fits well into current research results that reported mean FPS scores between 3.53 [4] and 3.83 [24]. While some studies were based on samples of nurses/nurses in training [4] or medical/physician assistant students [6], [28], [29], similar mean FPS scores were also found in other studies that were particularly based on population based samples [9], [10], [14]. The second index, the so-called stigma difference index, takes into account the responses of the evaluation of the normal weight vignette and represents the average difference between the mean evaluation of the obesity and the normal weight vignettes via the FPS. In other words, while the stigma index focuses on the assessment of attitudes towards obese individuals, the stigma difference index aims at the determination of different attitudes towards overweight and normal weight individuals. Using the stigma difference index has several important implications. In our study, two vignettes describing an individual varying in age, gender and body weight were used. In this context, the evaluation of the two different vignettes is based on these given characteristics (age, gender and body weight). In other words, the evaluation of the individual is not only based on the body weight but also on age and gender when using a vignette. Consequently, the stigma difference index that takes into account the different evaluations of overweight and normal weight individuals offers a methodical advantage insofar as the characteristics age and gender maintain constant while the characteristic body weight steps out more clearly in the direct comparison of the overweight and the normal weight vignettes. Thus, the stigma difference index takes up the idea that stigmatization is a contextual and relational process [30]. The stigma difference indices that were found in this study were similar to that found in another study evaluating the stigmatization of obese patients by health care professionals [27]. In future studies, the stigma difference index could serve as a useful measure for the magnitude of differences between negative attitudes towards obese and normal weight individuals.

In the measurement of attitudes towards overweight and obesity by using the FPS, previous studies introduced the cut-off value of 2.5 that classifies the study participants according to their positive/neutral or negative attitudes towards obese individuals [3], [14], [23]. In the present study, a smaller percentage of participants reported neutral or positive attitudes (0.9% vs. 3% to 13%) and a comparatively higher percentage reported negative attitudes (99.1% vs. 97% to 87%) towards obese people than in previous studies [5], [29]. This variation could be explained by the different sample structures of these studies including mainly assistant doctors, health care professionals, medical students, students of nutritional science or students of other disciplines. Maybe, these participants had more work-related education and expertise about the disease obesity than the general population, which may lead to less negative attitudes and reduced prejudice. Moreover, their occupational background may lead to more frequent contacts with obese patients, which could according to Allport’s intergroup contact hypothesis lead to a positive influence on the attitude towards obese people [31], [32]. On the other hand, the distribution of positive/neutral and negative attitudes towards obese people according to the cut-off of 2.5 was similar to the results of other studies that reported a lower proportion of subjects possessed neutral or positive attitudes towards obese individuals [6], [12].

On item level, our results confirmed the previous finding [6], [14] that all items of the FPS were rated significantly higher in the condition of the obesity vignette compared to the condition of the normal weight vignette. Furthermore, our results are consistent with previous studies [4], [29], [33] identifying the highest mean scores for the items that were related with food (dislikes food…likes food, undereats…overeats) or activity (having endurance…having no endurance, fast…slow). Altogether, the results indicate a high stability of the negative attitudes towards obese individuals in the general population.

### Analyses of variance

In accordance with previous studies, we observed significant group differences in the mean FPS scores for the variables age and BMI. With regard to age, the tendency observed in the present study that a higher age was associated with decreasing negative attitudes towards obese individuals was also found in previous studies. For example, Robinson et al. (1993) showed that participants aged 55 years and older possessed significantly less mean FPS scores and therefore less negative attitudes towards obese people than younger participants [9]. Similar results were obtained by Wolf (2012) [29]. Nevertheless, several studies did not find significant associations between age and negative attitudes towards obese individuals on a group level [6], [24], [34]. On the other hand, the study of Sikorski et al. (2013) that was based on a sample of health care professionals showed that a higher age was associated with a higher level of stigmatizing attitudes [33].

In the present study, overweight and obese participants reported significantly less negative attitudes towards obese individuals compared to underweight or normal weight participants. Thus, the finding that the level of negative attitudes towards obese people was higher in participants with a lower BMI was consistent with the results of many previous studies [9], [12], [14], [23], [24], [35]. In contrast to our results, other studies found that participants’ own body weight or BMI was unrelated to their scores on the FPS [4], [6], [34].

No significant differences in FPS mean scores on a group level were found for the variables gender, education, occupation, income, residence or overweight in personal history. In summary, the results of this study are widely consistent with previous research findings on the relationship between socio-demographic variables and the negative attitudes towards obese people. In agreement with previous study results, the trend that especially younger persons with a lower BMI possess relatively higher levels of stigmatizing attitudes towards overweight and obese people could be confirmed in this study.

### Reliability and validity

As in numerous previous studies [4], [9], [12], [14], [24], [35], a moderate to good reliability coefficient was obtained for the FPS in the present study (α = 0.79). However, in comparison with the results from Bacon et al. (2001), who found a Cronbach’s α = 0.91 for the FPS, the present reliability coefficient was somewhat lower. In total, the FPS can be considered as a reliable instrument for measuring negative attitudes towards obese individuals.

The evaluation of the correlations between the FPS and other construct-related instruments such as the scale for emotional reactions (SER) and the Perceived Devaluation and Discrimination Scale (PDD) yielded good evidence for the construct validity of the FPS. Consistent with previous studies [3], [36], [37] and as one would expect, a higher level of stigmatizing attitudes as measured by the FPS was significantly associated with stronger negative emotional reactions towards obese individuals (affective attitudinal dimension) and with a higher degree of agreement with common prejudices and negative attitudes of the general population towards overweight and obese people (cognitive attitudinal dimension). These findings correspond well with current research results. With regard to the cognitive dimension of negative attitudes, Puhl and Heuer (2009) found in their review that obese individuals encounter common prejudice and negative attitudes even amongst their close friends and family members [3]. The finding regarding to the affective attitudinal dimension that is associated with negative emotional reactions towards obese individuals is also supported by the finding that persons with normal weight and persons with physical disabilities were preferred as friends when compared to obese persons [37]. Moreover, another study showed that obese individuals were rather less preferred as (sexual) partners [36]. On the other hand and contrary to our initial expectation, no significant association between negative attitudes towards obese people as assessed by the FPS and the social distance to obese people as assessed by the Social Distance Scale (SDS) was found in our study. But, this finding fits in with the findings of O’Brien and colleagues who demonstrated that the negative attitudes towards obese individuals, per se, often do not adequately predict behavioral aspects such as actual acts of discrimination against obese individuals [38]. In other words, there seems to be a difference between the cognitive and affective attitudinal dimensions and the behavioral dimension. As the authors suggest, there is a need for improved questionnaire measures to better predict actual prejudiced behaviour [39].

Nevertheless, the present study offered the opportunity to explore the factorial structure of the FPS. In accordance with previous studies [9], [14], a one factor solution was strongly supported in our study. These results underline the assumption of the one-dimensional structure of the German short form of the FPS. In the future, additional exploration of the dimensionality of the short form of the FPS based on other samples would be desirable.

### Reference values

For the first time in Germany and following the recommendation of the authors of the short form of the FPS [10], we calculated reference values for the German version of the short form of the FPS based on an actual, large and representative sample (n = 1,657, 18–94 years). As we found significant age effects, the reference values in terms of percentage ranks were calculated not only for the total sample but also for different age groups. Furthermore, the reference values were developed not only for the FPS score (14–70) but also for the weighted FPS score (1–5) allowing the interpretation of individual test scores according to both evaluation modes. Altogether, the reference values might significantly contribute to the reliable and valid assessment of negative attitudes towards overweight and obesity via the FPS.

### Limitations

Limitations of this study refer to the limited comparability of studies using the FPS, particularly with regard to the different sample structures of studies. Many studies in this field were based on samples including specific occupational groups in health care and health care professionals only. In contrast, the present study was based on a sample that was representative for the German population. Nevertheless, the subjects of this study sample were, on average, slightly older and slightly more educated than the general population, which could limit the generalizability of the results. On the other hand, level of education did not seem to have a significant impact on the level of stigmatizing attitudes in our study. All measures were assessed via self-report. Another limitation of our study was that there were no indicators to control for social desirability, especially with regard to the telephone survey method used in this study. This may lead to an underestimation of the negative attitudes towards obese individuals. However, the mean FPS score in the present study was comparable to other studies and the problem of social desirability did not seem to have a significant impact on the results. An explanation for this could also be that the stigmatization of obese individuals seems socially more accepted than, for example, the stigmatization of ethnic minorities or gender. Furthermore, the expression of stigmatizing attitudes towards obese individuals could only be assessed after a verbal description of an obese person (overweight vignette). To what extent a visual presentation of an obese person, e. g. by a photo, would have affected the strength of the expressed stigmatization, however, was not assessable in this study. In addition, in the present study we only examined the negative attitudes towards obese individuals and not any discriminating behaviors. In this context, it remains unclear, however, whether and to what extent acts of discrimination were actually performed.

### Conclusions

In summary, this study demonstrated that the German short form of the FPS can be considered as a reliable and valid instrument for measuring the negative attitudes towards obese individuals. In addition, this study provides, for the first time in Germany, reference values for different age groups and for the total sample on the basis of a large, population-based and representative sample. Future investigations should address the calculation of reference values for other samples and settings including different occupational groups in healthcare. The further analyses of psychometric properties of the FPS should also be continued on an international level. Furthermore, the stigma difference index seems to be a promising approach that could be considered in future research on stigmatizing attitudes and discrimination. In our study, the stigma index and the stigma difference index were influenced by the variables age and BMI. In this context, further studies should take these potential influences into account. Finally, the short form of the FPS could be used more extensively in order to evaluate the effectiveness of anti-stigma campaigns or different interventions to reduce stigmatization of obese people.
